# Ethnic and functional differentiation of copy number polymorphisms in Tunisian and HapMap population unveils insights on genome organizational plasticity

**DOI:** 10.1038/s41598-024-54749-8

**Published:** 2024-02-26

**Authors:** Lilia Romdhane, Sameh Kefi, Nessrine Mezzi, Najla Abassi, Haifa Jmel, Safa Romdhane, Jingxuan Shan, Lotfi Chouchane, Sonia Abdelhak

**Affiliations:** 1 Genomics and Oncogenetics Laboratory (LR16IPT05), Institut Pasteur de Tunis, University of Tunis El Manar, Tunis, Tunisia; 2 Department of Biology, Faculty of Sciences of Bizerte, University of Carthage, Zarzouna, Tunisia; 3 Laboratory of Genetic Medicine and Immunology, Weill Cornell Medicine-Qatar, Education City-Qatar Foundation, Doha, Qatar; 4 Department of Genetic Medicine, Weill Cornell Medicine, New York, NY USA; 5 Genetic Intelligence Laboratory, Weill Cornell Medicine in Qatar, Education City, Qatar Foundation, Doha, Qatar

**Keywords:** Computational biology and bioinformatics, Genetics research, Genetic markers, Genomics, Population genetics

## Abstract

Admixture mapping has been useful in identifying genetic variations linked to phenotypes, adaptation and diseases. Copy number variations (CNVs) represents genomic structural variants spanning large regions of chromosomes reaching several megabases. In this investigation, the “Canary” algorithm was applied to 102 Tunisian samples and 991 individuals from eleven HapMap III populations to genotype 1279 copy number polymorphisms (CNPs). In this present work, we investigate the Tunisian population structure using the CNP makers previously identified among Tunisian. The study revealed that Sub-Saharan African populations exhibited the highest diversity with the highest proportions of allelic CNPs. Among all the African populations, Tunisia showed the least diversity. Individual ancestry proportions computed using STRUCTURE analysis revealed a major European component among Tunisians with lesser contribution from Sub-Saharan Africa and Asia. Population structure analysis indicated the genetic proximity with Europeans and noticeable distance from the Sub-Saharan African and East Asian clusters. Seven genes harbouring Tunisian high-frequent CNPs were identified known to be associated with 9 Mendelian diseases and/or phenotypes. Functional annotation of genes under selection highlighted a noteworthy enrichment of biological processes to receptor pathway and activity as well as glutathione metabolism. Additionally, pathways of potential concern for health such as drug metabolism, infectious diseases and cancers exhibited significant enrichment. The distinctive genetic makeup of the Tunisians might have been influenced by various factors including natural selection and genetic drift, resulting in the development of distinct genetic variations playing roles in specific biological processes. Our research provides a justification for focusing on the exclusive genome organization of this population and uncovers previously overlooked elements of the genome.

## Introduction

Copy number variations (CNVs) are genomic structural variants spanning large regions of chromosomes reaching several megabases^1–3^. In comparison to the reference genome, CNVs are generally observed as gain or loss of certain genomic region copies owing to their recombination mechanisms^4,5^. It has been demonstrated that 4.8–9.5% of the Human genome contributes to these structural variations^6^. In the Human genome, the ubiquitous feature of CNVs was underestimated until simulated series of efforts to identify and characterize them in diverse populations using genotyping arrays^7–11^ and recently using high-throughput sequencing^12–14^. These achievements resulted in the introduction of several terminologies including copy number polymorphisms (CNPs) characterized by a population frequency of at least 1%. They are also defined as common CNVs^7^.

CNVs exert their impact on phenotypes through the following mechanisms: by modifying the dosage of a gene, creating gene fusion, altering the distance of an entire gene from its regulatory elements, or altering the number of protein-coding exons within a gene^15–18^. Studies have demonstrated that CNVs contribute to approximately 18% of variation in gene expression, suggesting a pivotal role in determining complex traits^19^. Two models have been proposed to explain the association between CNVs and phenotypes. The first one involves CNPs and genes harboring such segments are essentially enriched for pathways and biological functions related to drug responses, immunity and sensory perception^20,21^. Consequently, these CNPs influence complex traits such as Crohn’s disease, glomerulonephritis in systemic lupus erythematosus and HIV-1/AIDS. Since CNPs could also occur in genes encoding drug-metabolizing enzymes, understanding their distributions is a key contributor to pharmacogenomics screenings^22^. A growing body of evidence reveals the essential role of CNVs as drivers of phenotypic diversity, evolution and adaptation in humans^12,16,23^. For example, it has been shown that copy number polymorphisms in *AMY1* amylase gene has been linked to preferences for high starch diet while deletions or insertions in the *APOBEC3b* gene have been associated to differences in malaria susceptibility^23,24^. The second model encompasses rare and highly penetrant CNVs that are essentially involved in genomic disorders.

Traditionally, studies in human population genetics have predominantly concentrated on Single Nucleotide Polymorphisms (SNPs) to infer demographic shifts such as resulting from bottlenecks or founder events and to examine gene flow due to migration. However over the past two decades, there has been a growing body of research utilizing CNVs that serve as insightful markers in population genetics investigations and confirming their functional potential and their evolutionary relevance. These structural genomic changes contribute to genetic diversity across populations offering a unique perspective on the evolutionary history and genetic differentiation of human groups. The population-specific CNVs reflecting historical migration patterns and isolation events has become a noteworthy aspect of this research. Additionally, analyzing the frequency distribution of CNVs could provide information about demographic processes, founder effects within populations and insights into local adaptation^2,12,13,25–28^.

Tunisia, located in North Africa with a population of 11 million inhabitants, has a diverse and rich demographic history. According to evidence from mitochondrial DNA, a group that originated from sub-Saharan Africa occupied Tunisia over 20,000 YBP^29^. By 15,000 YBP, Ibero-Maurisans exhibiting anatomical similarities to the European Cro-Magnons, appear in the region^30^. The wet climate of the Sahara, before 9000 years ago, allowed the local Tunisian population to coexist and mix with migrants from sub-Saharan Africa^31^. The Capsian, a community of proto-Mediterraneans migrated and expanded extensively throughout present-day Tunisia and could admixed with pre-existing populations^32^. Since 4000 YBP, Berbers have migrated through North Africa^33^. In historical times, the Tunisian region witnessed multiple invasions and migratory waves of ethnic groups and allogenic populations mainly from Europe and the Middle East. These included Phoenicians, Greeks, Romans, Vandals, Byzantines, Arabs, Spanish, Ottomans, Andalusians and French^34^. The majority of these groups have left their mark on the present-day Berber population. Nonetheless, the most significant change in the past few centuries resulted from the arrival of Arabs and Bedouins, leading to the conversion of a considerable portion of the original population to Islam and Arab culture^34^. Like contemporary Arab populations, the Tunisian one is featured by high rates of endogamy and consanguinity that could reach 98% and 38% respectively, especially in rural areas^35,36^. Both social cultural features and historical events have impacted its genetic properties and the distribution of functional variants in relation to health and diseases^36,37^.

In our previous study, we reported the first CNV map of the Tunisian population including 1083 segments and spanning 61.443 Mb of the genome by merging CNVRs (Copy Number Variation Regions) and CNPs^38^. Functional annotation contributed to improving our understanding on this unstudied kind of variation in the Tunisian population revealing that some CNV genes are involved in biological pathways relevant to public health^38^. Additionally, the population stratification based on a correlation frequency analysis suggested an European contribution in the genetic background of the Tunisian population^38^. In this present work, we conducted a population genetics investigation using the CNP makers previously identified in the Tunisian population. Thus, we described and compared the CNP features in the Tunisian population and 11 HapMap populations highlighting that CNV diversity differs at the population level. We performed CNV sharing analysis to identify CNP patterns of genomic distribution across populations. In addition, we assessed population differentiation by calculating FST using allele frequency. A detailed population stratification analysis with a principal component analysis and the STRUCTURE algorithm highlighted that population structure can be detected by CNP data. Furthermore, we performed a population-specific analysis and discovered several CNP candidates exhibiting notable divergent signals that might be subjected to selective pressures. The results extend our understanding on the copy number variation in the human genome and could provide an essential framework for grasping the genomic differentiation of complex traits among various ethnic groups.

## Results

### CNP distribution in the studied populations

**Figure 1:**
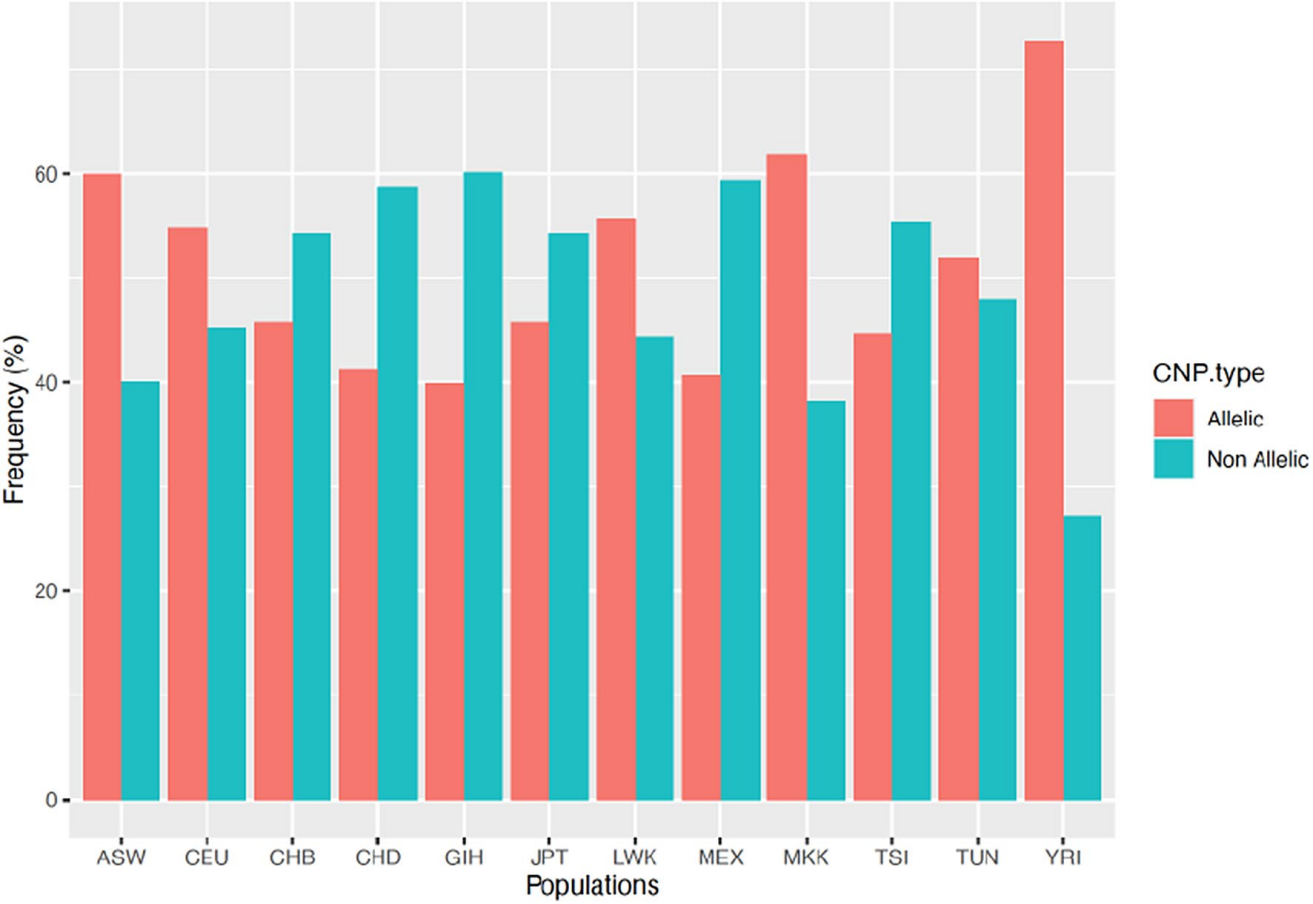
Allelic distribution in the Tunisian and HapMap populations of the 1279 studied CNPs.

Following the application of quality filters for the called CNPs across the populations, the sub-Saharan African populations showed the highest amount of diversity characterized by the highest proportions of allelic CNPs identified. The Asian GIH population represented the least diverse group (Fig. 1, Supplementary Table 1). Within the African populations, Tunisia demonstrated the least diversity with 665 genotyped CNPs (55.99%). The distribution of these CNPs in Tunisia showed a significant difference compared to all the populations except for of LWK and CEU (Fig. 1, Supplementary Table 1). The Tunisian population displayed the lowest the least prevalence of deletions among the African populations. Similarly and as stated earlier, the distribution of these loci in the Tunisian population showed a significant difference except for the LWK and CEU populations (Supplementary Table 2).

### Frequency features of genotyped CNPs in the studied populations

In all the studied populations, the frequency of allelic CNPs ranged from singleton (CNP identified in one individual) to completely being fixed and reaching 100% (Supplementary Table 3). In the Tunisian population, the median frequency is assessed to 4.25% indicating that half the 665 allelic CNPs would have a frequency less than 4% and thus categorizing them as rare. This value was the lowest among the studied populations. The frequency distribution was similar to that of MKK, YRI and CEU populations (Supplementary Table 3). Furthermore, there is a high proportion of rare CNPs with frequencies ranging from 1 to 10% in the following populations: MKK (65.57%), ASW (64.80%), CEU (63.77%) and TUN (63.46%) (Table 1). CNPs with frequencies exceeding 50% are less frequent. The distribution of CNP frequencies among the different frequency categories in the Tunisian population is significantly different from those of ASW, LWK, YRI and GIH populations (Table 1). We performed the same analysis taking into account the segment type (Supplementary Tables 4–6). Similarly, we noticed a high rate of deletions with frequencies less than 10% with the highest proportion noted among the Tunisian genome (75.77%) (Supplementary Table 4). Regarding the duplications, we observed that almost all of them have frequency values less than 10%. The distribution is similar to that of all the studied populations (Supplementary Table 5). Similarly, no significant difference has been found after comparing the Tunisian population with each of the HapMap populations regarding mixed loci (Supplementary Table 6).

**Table 1:** The number of loci and the corresponding percentage with varying population frequencies.

Continent	Population	Frequency class				Chi-square test FDR (p-value < 0.05)
		[0%, 10%[	[10%, 50%[	[50%, 100%[	100%	
Africa	ASW	497 (64.80%)	193 (25.16%)	76 (9.90%)	1(0.13%)	**0.0154**
	LWK	421 (59.13%)	204 (28.65%)	80 (11.24%)	7 (0.98%)	**0.0181**
	MKK	518 (65.57%)	189 (23.92%)	77 (9.75%)	6 (0.76%)	0.0716
	YRI	618 (48.32%)	225 (17.59%)	82 (6.41%)	6 (0.47%)	**0.0109**
	TUN	422 (63.46%)	141 (21.20%)	97 (14.58%)	5 (0.75%)	**–**
Europe	CEU	447 (63.77%)	160 (22.82%)	94 (13.41%)	0 (0%)	0.1584
	TSI	334 (58.49%)	143 (25.04%)	90 (15.76%)	4 (0.70%)	0.3977
America	MEX	293 (56.35%)	134 (25.77%)	88 (16.92%)	5 (0.96%)	0.1473
Asia	CHB	346 (59.16%)	146 (24.96%)	90 (15.38%)	3 (0.51%)	0.4197
	CHD	296 (56.06%)	135 (25.57%)	90 (17.05%)	7 (1.33%)	0.1137
	JPT	338 (57.78%)	153 (26.15%)	91 (15.56%)	3 (0.51%)	0.1970
	GIH	266 (52.16%)	160 (31.38%)	80 (15.69%)	4 (0.78%)	**0.0017**

Significant values are in bold.

### Population differences in the CNP integer copy numbers

**Figure 2:**
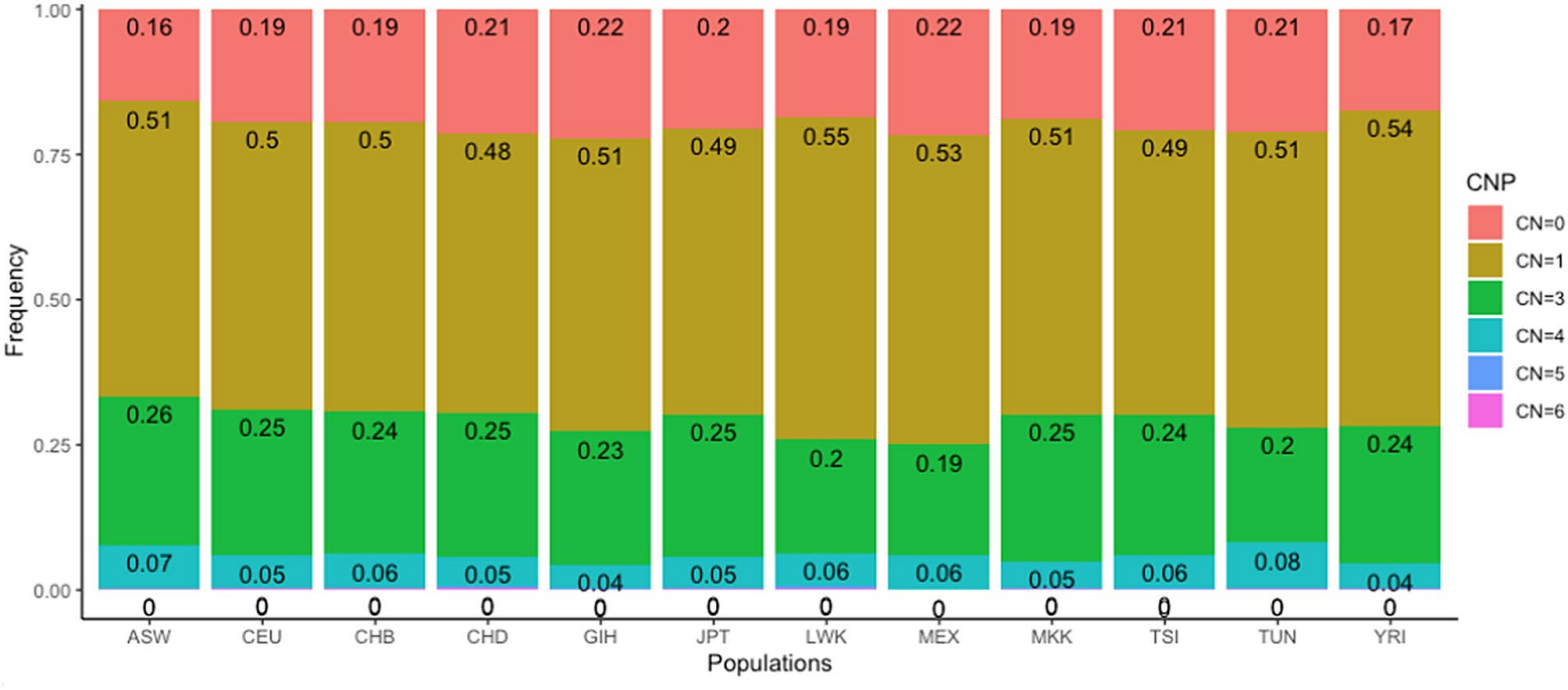
Copy number state for all loss and gain CNP loci across the studied populations.

Our focus on the integer copy number of the CNPs identified in Tunisia aimed to highlight any population difference. Across all the populations, heterozygous deletions (CN = 1) were the most prevalent with proportions values ranging from 48.01% (CHD) to 55.43% (LWK) while amplifications (CN = 5 and CN = 6) were the least common (Fig. 2). Given that the Tunisian population is characterized by high rates of consanguinity, reaching up 38% and even 65% in certain regions^39–41^, we expected that the Tunisian genome would be featured by the highest proportions of homozygous segments. This was indeed true for homozygous duplications (CN = 4) with the highest value recorded at 7.89%. Nevertheless, the homozygous deletions (CN = 0) were the most prevalent in the GIH population (22.12%), MEX (21.6%), CHD (21.42%) followed by the Tunisian population (21.08%) (Fig. 2).

To elucidate relationships of the Tunisian population with the other populations, CNPs that were shared between populations were assessed. The Tunisian population displayed the highest amount of shared allelic CNPs with the Africans, followed by the Europeans (Supplementary Table 7). Furthermore, **153** CNPs were allelic and observed in all the studied populations qualifying them as shared common CNPs. No allelic CNPs were exclusively observed in the Tunisian population that were absent in all the other populations.

### Tunisian high-frequent CNPs

We performed pairwise comparison in order to identify differences in CNP frequencies between the Tunisian population and the eleven other studied groups (Supplementary Table 7). This analysis performed on the 1279 CNPs revealed a similarity in CNP profiles between the Tunisian population and those of European descent, MEX and GIH with lowest count of significantly differing CNPs (Supplementary Table 7).

**Figure 3:**
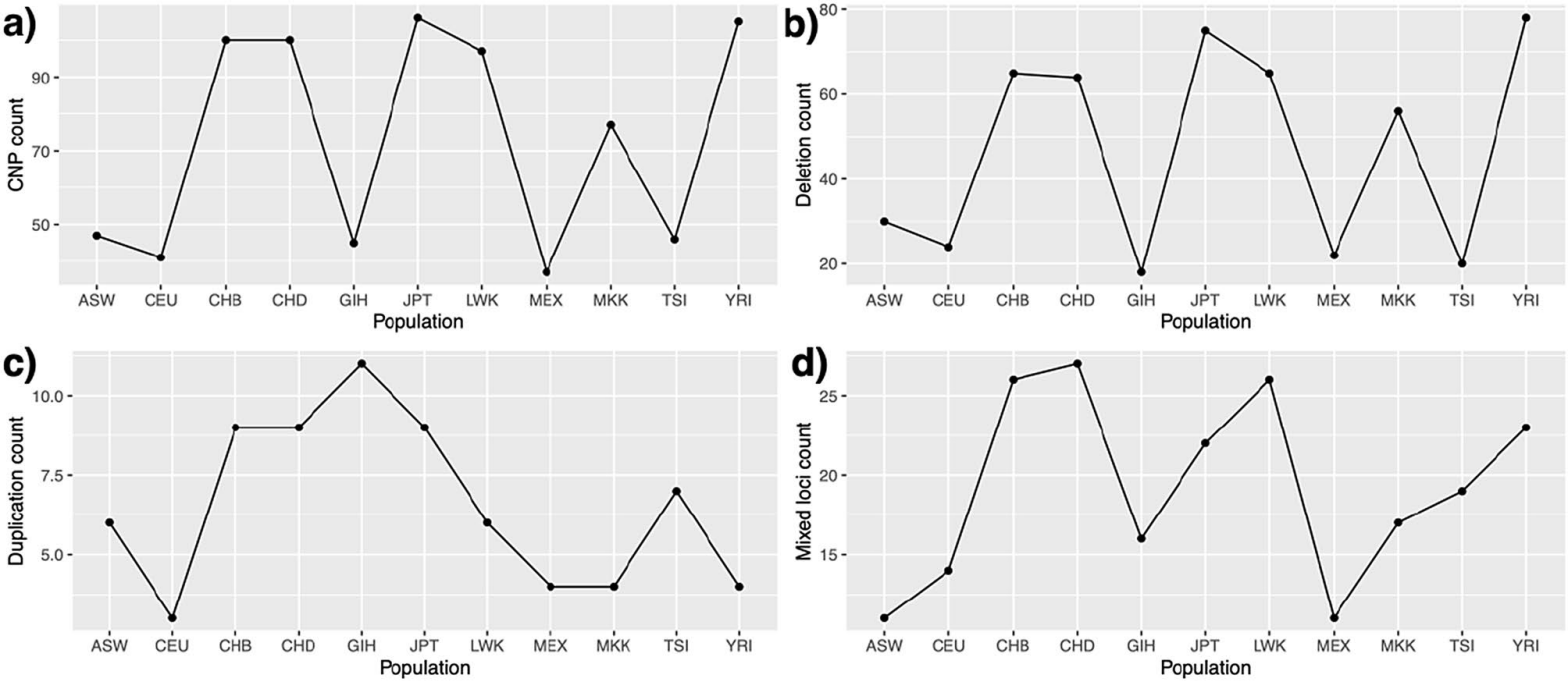
Counts of Tunisian high-frequent CNPs compared to all populations: (**a**) All Tunisian high-frequent CNPs. (**b**) Tunisian high-frequent deletions. (**c**) Tunisian high-frequent duplications. (**d**) Tunisian high-frequent mixed loci.

We identified **162** allelic CNPs with significantly higher frequency in Tunisia than in all the other populations (herein denotes as Tunisian high-frequent CNPs). These were classified into **99** deletions, **13** duplications and **50** mixed loci. The distribution of the Tunisian high-frequent CNPs followed the same pattern as previously noted with lower counts with populations of European, Mexican and Indian descents (Fig. 3).

### Functional annotation and health impact of CNPs

The **162** Tunisian high-frequent CNPs are overlapping with **138** RefSeq genes (85 protein coding genes, 53 non-coding genes). Among the coding genes, 54 harbor 31 CNPs in their coding sequences. Within these coding genes, 27 are completely deleted or duplicated by 17 CNPs. The OMIM database were queried using the protein coding genes. In order to identify genes potentially linked to diseases, our focus was on functional deletions or duplications that affect exons either overlapping the entire genes or that potentially disrupting the protein translation frame. Seven genes are known to cause 9 Mendelian diseases and/or phenotypes (Table 2). These genes harbor 6 CNPs. The ACMG classifications shows that the CNP799 deletion is classified as likely pathogenic and the four other duplications as of uncertain significance. The mixed locus CNP2270 is annotated as of uncertain significance if it is a duplication and likely pathogenic if it is a deletion. The other mixed segment (CNP928) could be considered as of uncertain significant if it is a duplication and pathogenic if it is a deletion (Table 2). Compared with our previous study on CNVs, the Tunisian population seems to be at higher risk of developing 9 out of 155 (6%) of the reported diseases due to deletions or duplications of genomic regions^38^.

**Table 2:** Tunisian high-frequent CNPs affecting OMIM Mendelian disease and phenotype genes and corresponding frequencies.

OMIM phenotype (OMIM ID)	Inheritance	Gene	CNP ID	Position	Length	Type	Location	ACMG class	TN	ASW	LWK	MKK	YRI	CEU	TSI	MEX	CHB	CHD	JPT	GIH
Molybdenum cofactor deficiency B (252160)	AR	*MOCS2*	CNP799	5:52404519–52409439	4920	DEL	txStart-intron1	4	0.58	0.52	0.62	0*	0.38*	0.41	0.11*	0.3	0.15*	0.33*	0.35*	0*
Resistance to Malaria (611162); Susceptibility to systemic lupus erythematosus (152700)	Unknown; AD	*FCGR2B*	CNP118	1:161511410–161639559	128,149	DUP	txStart-intron1	3	1	1	1	1	1	0.99	1	0*	0.99	1	0*	0*
Immunodeficiency 20 (615707)	AR	*FCGR3A*	CNP118	1:161511410–161639559	128,149	DUP	txStart-txEnd	3	1	1	1	1	1	0.99	1	0*	0.99	1	0*	0*
Premature ovarian failure 12 (616947); Spermatogenic failure 15 (616950)	AR;AR	*SYCE1*	CNP1670	10:135328663–135377278	48,615	DUP	intron1-txEnd	3	0.14	0.22	0.13	0.16	0.16	0.03	0.05	0.02	0.02*	0.03	0.03	0*
Koolen-De Vries syndrome (610443)	AD	*KANSL1*	CNP2269	17:44165801–44364214	198,413	DUP	txStart-intron4	3	0.35	0.26	0.01*	0.07*	0*	0.35	0*	0.34	0*	0.01*	0.01*	0*
Developmental and epileptic encephalopathy 96 (619340)	AD	*NSF*	CNP2270	17:44401067–44752300	351,233	MIX	txStart-intron9	3 if duplication and 4 if deletion	0.89	0.71	0.67*	0.85	0.70*	0.74	0.68*	0.75	0.84	0.85	0.83	0.85
Susceptibility to carbamazepine-induced hypersensitivity syndrome (608579)	Unknown	*HLA-A*	CNP928	6:29837192–29921127	83,935	MIX	txStart-txEnd	3 if duplication and 5 if deletion	0.95	0*	0.99	0.99	0.87	0.99	0.94	1	0.91	0.95	0.98	0.94

*AR* Autosomal Recessive, *AD* Autosomal Dominant, *txStart* Transcript Start, *txEnd* Transcript End.

*Fisher test p-value FDR < 0.05

We also checked if Tunisian high-frequent CNPs overlap with pharmacogenes. Ten (10) genes overlapped with 10 CNPs are shown to be involved in pharmacodynamics of 28 chemical compounds in 20 diseases including cancers (breast, testicular, leukemia), hypertension, severe disorders of the skin (Stevens-Johnson syndrome, toxic epidermal necrolysis), nervous diseases (schizophrenia ad neuromyelitis optica), immune system diseases (acquired immunodeficiency syndrome, drug reaction with eosinophilia and systemic symptoms) (Supplementary Table 8).

### Population stratification and genetic relationships

**Figure 4:**
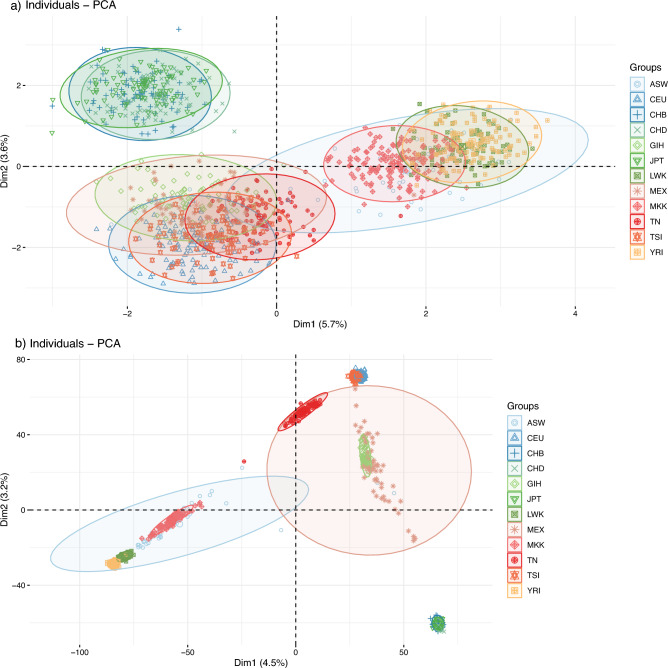
Principal component analysis (PCA) inferring population stratification of Tunisian and HapMap samples based on biallelic CNPs (**a**) and genome-wide SNP markers (**b**).

Taking into account the CNP map, we assessed the fine-scale population genetic structure and corresponding relationships. We conducted the principal component analysis (PCA) with the 1279 CNPs. The PCA showed different clusters for populations with various ancestries. Populations were separated along Dim1 (Sub-Saharan Africans vs non-Sub-Saharan Africans) and Dim2 (East Asians vs Africans, Europeans and Americans) (Fig. 4). Therefore, the two principal components (Dim1 and Dim2) indicated three clusters: the first comprised East Asian populations encompassing the Chinese and the Japanese. The second one is represented by the Sub-Saharan Africans (MKK, YRI, LWK and ASW). The third cluster included Tunisian, Italian, French and unexpectedly Indian and Mexican populations (Fig. 4a). The Tunisian population clustered between the Sub-Saharan Africans and the Europeans indicating that the CNP profile of the Tunisians shows similarity with those of European ancestries. This observation substantially suggests that the CNP genetic structure of the Tunisians differs from that of Sub-Saharan African and East Asians hinting at genetic admixture of the Tunisians with the Europeans. Moreover, this result suggests also that the CNP profile of the Gujarati Indians is different from those of Chinese and Japanese and resembles that of individuals with European ancestries and Tunisians. Using a set of 341,901 SNPs of the 1093 individuals of this study, we validated again our observations of the distinctiveness of the Tunisian population forming a perfect cline between European populations and Sub-Saharan populations in addition the relatedness with Mexican and Indian populations (Fig. 4b).

### Population differentiation analysis

**Figure 5:**
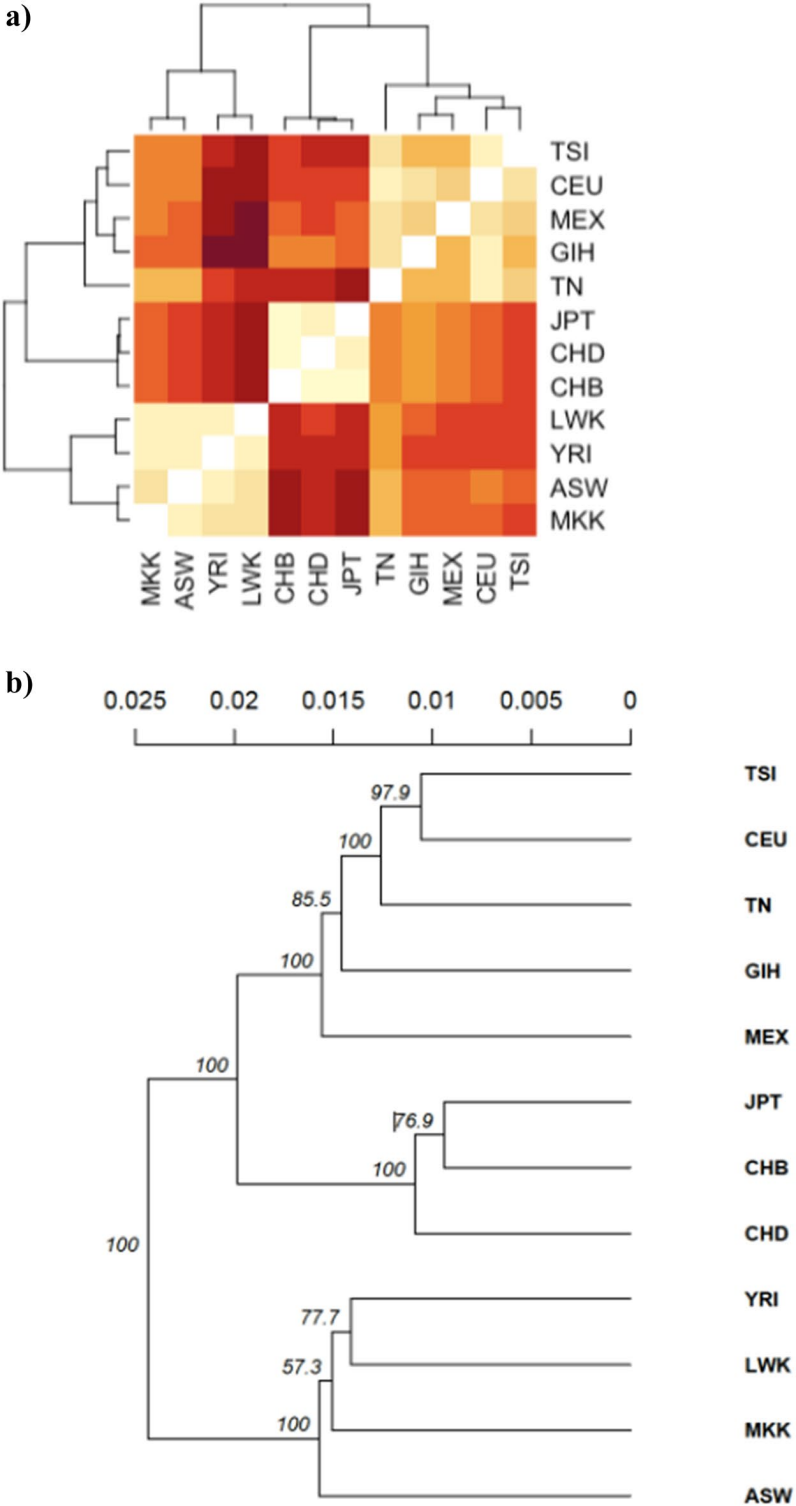
Genetic differentiation extent between Tunisian and each of the HapMap populations. (**a**) The pairwise FST analysis shows more closeness of Tunisians with European populations compared to African. This finding is in agreement with our clustering analysis. (**b**) Phylogenetic tree of the studied ethnic group constructed by UPGMA and in agreement with the PCA results.

We used the unbiased *Fst* statistic to measure population differentiation. The pairwise population *Fst* values were computed after CNP allele frequencies among the Tunisian population and the other 11 HapMap populations (Fig. 5a). The observed values ranged between 0.02 and 0.08. The lowest level of differentiation was observed with the European CEU population (0.022), followed by the TSI (0.036), GIH (0.038) and the MEX (0.042), indicating a weak genetic differentiation when comparing Tunisia to these other HapMap populations. However, the highest divergence was observed with the Japanese JPT population (0.083) thus suggesting a moderate level of differentiation. The Fst clustering pattern values coincided with the results of the PCA identifying three distinct clusters.

Based on the genetic distance, we built a phylogenetic tree with a topology revealing three major clades. This outcome aligns in agreement with the PCA results, albeit placing a last common ancestor (LCA) of the Asian group closer to the TN-CEU-TSI-GIH-MEX group rather than the expected African one (Fig. 5b).

**Figure 6:**
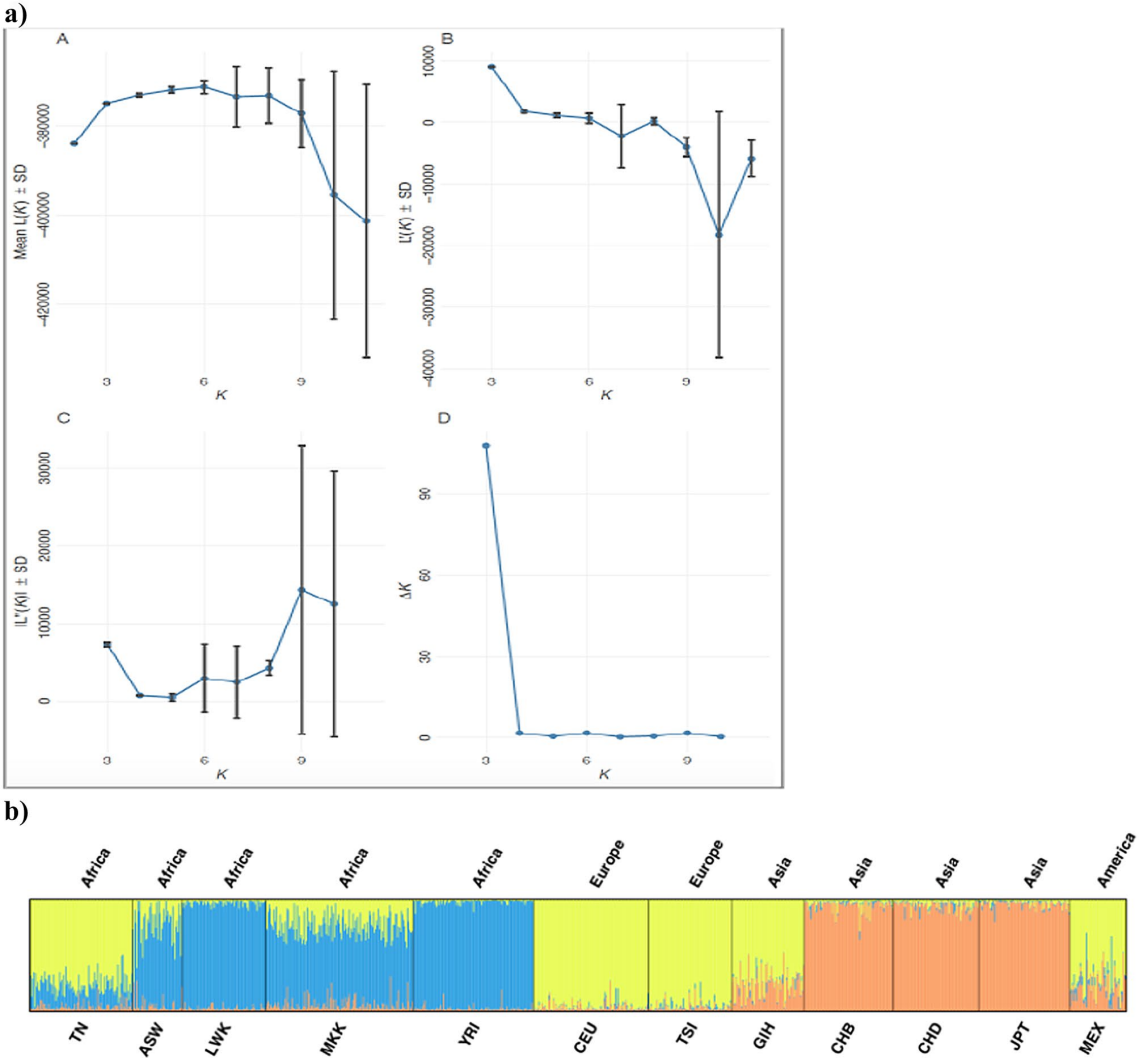
Population stratification using STRUCTURE. (**a**) K-value selection (Evanno method) graphs from STRUCTURE output. Mean posterior probability (A), change of mean posterior probabilities rate (B), second order rate of change of mean posterior probabilities (C) and *ΔK* values^42^ (D) from POPHELPER for STRUCTURE runs for *K* = 2–11. (**b**) Individual ancestry proportion estimates of Tunisian individuals using STRUCTURE. A K = 3, a major European component (yellow) accounts for ancestry proportion of 76% with less from Africa (Blue) and Asia (Orange).

To assess the discriminatory the power of the 1279 CNPs across populations of different ancestries, 24 STRUCTURE runs were performed using 2–11 groups (K = 2–11). The Evanno delta K method, employed to estimate the number of possible sub-populations, suggested a maximum of 3 populations (Fig. 6a). Considering K = 3, our observations revealed a predominant European ancestry contribution (76%), followed by the African (20%) and the Asian (3%) components (Fig. 6b) thus supporting both the PCA analyses. The inferred ancestry of each individual of this K value provided in further details (Supplementary Table 9). This observation reflects that the Tunisian population represent a mosaic of various populations, indicative of distinct gene flows. Additional STRUCTURE plots for the remaining putative number of ancestors (K) are provided (Supplementary file 1).

### Selection signatures among Tunisians

We performed a selection signature analysis within the Tunisian population using the pairwise Fst statistics by comparing Tunisians with each of the HapMap populations, respectively. This analysis aimed to identify CNPs or CNPs-harbored genes potentially subject to positive selection. The top 1% loci of the Fst values of each pair were listed as candidate regions of adaptation among Tunisians (Supplementary Table 10). We identified 47 CNPs as candidate loci for selection signature overlapping by 84 RefSeq (Supplementary Table 10). Notably, 30 of these candidate loci were Tunisian high-frequent (Supplementary Table 10). GO annotation revealed significant functional and biological processes related to receptor pathway and activity as well as glutathione metabolism (Table 3). In addition, the 53 coding gene pathway analysis revealed 16 highly enriched pathways of potential relevance to health such as drug metabolism, infectious diseases and cancers of which 5 have been already identified in our previous study^38^ (Table 3). Among the CNPs identified as candidates for selection signature, 11 are overlapping with 12 RefSeq genes known to cause Mendelian diseases/phenotypes (Table 3). Our previous LD analysis of CNV with neighboring SNPs revealed that 12 of the selection candidate CNPs exhibit high linkage disequilibrium (LD) (> 0.5)^38^ (Supplementary Table 10).

**Table 3:** Functional annotation of candidate genes harboring CNPs with signal of selection.

GO category	GO term	GO annotation	Gene count	Genes	Fold enrichment	FDR
Biological Process	GO:0006749	Glutathione metabolic process	5	*GSTM2, GSTM1, GSTT4, GSTT2, GSTT2B*	50.16	5.02 × 10^−4^
Biological Process	GO:0007166	Cell surface receptor signaling pathway	7	*FCGR3A, FCGR3B, MRGPRX1, LILRB3, FCGR2B, FCGR2C, SIRPB1*	10.82	3.4 × 10^−3^
Biological Process	GO:0050776	Regulation of immune response	4	*FCGR3A, FCGR3B, FCGR2B, FCGR2C*	43.86	6 × 10^−3^
Molecular Function	GO:0004364	Glutathione transferase activity	5	*GSTM2, GSTM1, GSTT4, GSTT2, GSTT2B*	83.37	2.64 × 10^−5^
Molecular Function	GO:0019864	IgG binding	4	*FCGR3A, FCGR3B, FCGR2B, FCGR2C*	163.70	6.32 × 10^−5^
Molecular Function	GO:0004888	Transmembrane signaling receptor activity	6	*FCGR3A, FCGR3B, MRGPRX1, CHRFAM7A, FCGR2B, FCGR2C*	14.29	1.45 × 10^−3^
KEGG Pathway	hsa04380	Osteoclast differentiation	6	*FCGR3A, FCGR3B, LILRB3, FCGR2B, FCGR2C, SIRPB1*	22.62	1.2 × 10^−4^
KEGG Pathway	hsa05204	Chemical carcinogenesis—DNA adducts	5	*GSTM2, GSTM1, UGT2B17, GSTT2, GSTT2B*	34.97	1.2 × 10^−4^
KEGG Pathway	hsa00982	Drug metabolism—cytochrome P450	5	*GSTM2, GSTM1, UGT2B17, GSTT2, GSTT2B*	33.52	1.2 × 10^−4^
KEGG Pathway	hsa00980	Metabolism of xenobiotics by cytochrome P450	5	*GSTM2, GSTM1, UGT2B17, GSTT2, GSTT2B*	30.94	1.2 × 10^−4^
KEGG Pathway	hsa00983	Drug metabolism—other enzymes	5	*GSTM2, GSTM1, UGT2B17, GSTT2, GSTT2B*	30.16	1.2 × 10^−4^
KEGG Pathway	hsa00480	Glutathione metabolism	4	*GSTM2, GSTM1, GSTT2, GSTT2B*	33.87	1.1 × 10^−3^
KEGG Pathway	hsa04145	Phagosome	5	*FCGR3A, FCGR3B, HLA-A, FCGR2B, FCGR2C*	15.88	1.1 × 10^−3^
KEGG Pathway	hsa01524	Platinum drug resistance	4	*GSTM2, GSTM1, GSTT2, GSTT2B*	26.45	2 × 10^−3^
KEGG Pathway	hsa05207	Chemical carcinogenesis—receptor activation	5	*GSTM2, GSTM1, UGT2B17, GSTT2, GSTT2B*	11.38	2.95 × 10^−3^
KEGG Pathway	hsa05150	Staphylococcus aureus infection	4	*FCGR3A, FCGR3B, FCGR2B, FCGR2C*	20.11	3.35 × 10^−3^
KEGG Pathway	hsa05418	Fluid shear stress and atherosclerosis	4	*GSTM2, GSTM1, GSTT2, GSTT2B*	13.89	8.9 × 10^−3^
KEGG Pathway	hsa05225	Hepatocellular carcinoma	4	*GSTM2, GSTM1, GSTT2, GSTT2B*	11.49	1.4 × 10^−2^
KEGG Pathway	hsa05152	Tuberculosis	4	*FCGR3A, FCGR3B, FCGR2B, FCGR2C*	10.72	1.56 × 10^−2^
KEGG Pathway	hsa05208	Chemical carcinogenesis—reactive oxygen species	4	*GSTM2, GSTM1, GSTT2, GSTT2B*	8.66	2.62 × 10^−2^
KEGG Pathway	hsa05140	Leishmaniasis	3	*FCGR3A, FCGR3B, FCGR2C*	18.80	2.74 × 10^−2^
KEGG Pathway	hsa04666	Fc gamma R-mediated phagocytosis	3	*FCGR3A, FCGR3B, FCGR2B*	14.93	4 × 10^−2^

## Discussion

The ubiquitous CNV in the human genome largely play a part in phenotypic divergence and impacts disease predisposition and overall health^12,13,43^. The complex history and numerous inflows of populations in Tunisia and the environmental pressure have collectively shaped the unique genetic diversity observed in the Tunisian population.

In our study, we found that approximately half of the CNPs previously identified by MacCarroll et al., were not polymorphic in all the Asian, the TSI and MEX populations^7^. The African populations showed the highest amount of diversity with the greatest proportions of allelic CNPs which is in firm concurrence with the recent findings where the distribution of structural variations across samples matched expectations based on human demographic history^13^. In addition, it has been shown that African and African-American individuals exhibited the greatest diversity while the Europeans demonstrated the least diversity^13^. Our results emphasize the importance of characterizing CNPs in different populations. Furthermore, we highlighted that the deletion count is higher than that of duplications. However, the size of duplication is much larger than that of deletion (Supplementary Tables 11 and 12). This observation aligns with our previous reported when establishing the CNV map of the Tunisian population and mirrors a similar pattern detected in other studies employing different arrays, algorithms and methods^25,38,44–46^.

Regarding CN state, it was imperative to evaluate the zygosity of all identified CNPs to accurately analyze genome changes. In all the populations and among the CN states, heterozygous deletions (CN = 1) were the most prevalent with proportions ranging from 48.01% (CHD) to 55.43% (LWK), with Sub-Saharan-Africans, Tunisians, and Mexicans showing the highest values. The homozygous deletions (CN = 0), were more prevalent among the Asian cluster, followed by the group formed by Tunisian–European–Mexican and finally the African cluster showing the lowest frequency. This observation joins not only the enrichment of healthy individuals’ genomes in deletions but also suggests that these deletions could be common polymorphisms depicting ancestral mutations in LD (r^2^ measure) with neighboring SNP that occurred before humans migrated from Africa to Europe and Asia^13,47^. Moreover, we found that homozygous deletions were more prevalent in Asian genomes as it has also been shown in a previous study although this did not align with the worldwide distribution of consanguinity rates^13,48^. Genome accumulation did not show any specific continental pattern as the most proportions of heterozygous duplication (CN = 3) (> 0.24%) are identified among the following populations: ASW, MKK, CEU, TSI, CHB, CHD and JPT. The homozygous duplications (CN = 4) were encountered with the highest proportion among Tunisians followed by the ASW population suggesting a unique genome founder state which was not found in any genome of the other populations. Accordingly, it has been suggested that the CN state could be an indicator of the populations losing genomic regions when compared to Africa^26^. Conducting a comprehensive analysis of the five CN state enables the determination of the overall loss and gain of a genome across diverse populations. This approach highlights the influence of distinct evolutionary dynamics on the genomes and serves as a critical instrument to investigate the genomic evolutionary drift within populations.

It is essential for healthcare providers and patients to comprehend the frequency of significant genetic variations that impact both diseases and drug effectiveness and potential harm. Our functional analysis of Tunisian high-frequent CNP uncovers interesting findings in relation with health. Tunisian high-frequent CNPs overlapped with coding sequences of genes involved in Mendelian diseases/phenotypes suggesting that the Tunisian population is at risk of developing such traits. However, it is worth recalling that all the individuals in our analysis are healthy. Consequently, even though we have observed a significant number of deletions and duplications in coding sequences, including homozygous complete genes losses, it is not possible to rule out the potential involvement of the carried CNV in causing a disease in the absence of comprehensive prospective phenotypic data^38^. We also noticed that CNPs with high frequency in the Tunisian population overlapped with pharmacogenetic genes. It has been demonstrated that CNVs in some pharmacogenes contribute to drug efficacy and toxicity^49,50^. The frequencies of pharmacogenetic alleles can significantly differ between ethnic groups thus impacting the drug variability between individuals and between populations^51,52^. GSTM1 belongs to the Glutathione S-transferases (GSTs) enzyme superfamily which is involved in the carcinogen detoxification, environmental toxins and therapeutic chemicals. Complete gene deletions of *GSTM1* are relatively common and individuals with European ancestry seems to be homozygous for this gene deletion (50%) more frequently than Asian (22%) and African (27%)^53,54^. This difference in gene frequency across different ethnic populations is attributed to their various evolutionary histories and method selection^55^. In our analysis, deletion of this genes reaches higher frequencies (92%) in Tunisians (51% for the homozygous deletion et 41% for the heterozygous deletion), the French (CEU) and the Chinese (CHB) populations and seems to be absent in the Italian (TSI) and Chinese (CHD) populations. A previous study reported a high prevalence of the null GSTM1 genotype with a distinct distribution between the North, the Center and the South of Tunisia but without any distinction between Cosmopolitans, Arabs and Berbers^56^. As *GSTM1* is crucial in the detoxification of external toxins from the body, individuals with deleted copies of this gene are at a higher risk for developing different types of cancer and other multifactorial diseases as well as drug related toxicities. In Tunisia, neurological diseases are frequent in Tunisia and CNVs could be a risk factor for their expression^38,57^. In Center Tunisia, where the prevalence of Parkinson disease is 22/100,000, the *GSTM1* null genotype increases the disease risk (OR = 5.45, 95% IC 2.90–10.30, p-value = 10^−6^) that could be amplified by the presence of the *GSTT1* null genotype^58^. Furthermore, Tunisian individuals harboring a homozygous *GSTM1* gene deletion exhibit a 3.8 fold increased risk of developing epilepsy (OR = 3.8, 95% IC 2.15–4.78.30, p-value < 10^−6^)^59^. Bipolar disorder seem be expressed due the combination of the two null genotypes of *GSTM1* and *GSTT1* (OR = 2.96, 95% CI (1.26–7.03), p = 0.005)^60^.

Regarding cancers, bladder cancer is increased to fourfold in North Tunisia among tobacco consumers (OR = 4.35, 95% IC 1.78–10.77, p-value = 6 × 10^−4^)^61^, nasopharyngeal carcinoma to twofold (Odds Ratio = 2.12, [0.64–4.7])^62^ and acute lymphoblastic leukemia to twofold (OR = 2.05, 95% IC 1.05–3.79, p-value = 3.1 × 10^−2^)^63^ in presence of *GSTM1* null genotype. The same genotype could be associated to other diseases such asthma (OR = 2.35, 95% IC 1.30–4.27, p-value = 2 × 10^−3^)^64^. Homozygous deletion of this gene is also clearly associated with the chronic obstructive pulmonary disease (OR = 1.58, 95% IC 1.06–2.35, p-value = 0.02) but after the prominent chronic obstructive pulmonary disease risk factors were excluded the associations had disappeared. It was suggested that the two copy *GSTM1* deletion seemed not to be an independent risk factor for the disease^65^. The risk related to the same genotype was twofold in the development of endometriosis (OR = 2.37, 95% IC 1.42–3.96, p-value = 10^−3^) and increased in combination with the *GSTT1* null genotype (OR = 8.42, 95% IC 2.93–24.14, p-value = 2 × 10^−5^)^66^.

In our present study, we observed that the Tunisian population could be at risk of developing the susceptibility to carbamazepine-induced hypersensitivity syndrome. In a recent work on evaluating the contribution of carbamazepine, an anti-epileptic drug, to a mild hepatotoxicity, the *GSTM1* null genotype was found to be associated with elevated levels of both alanine aminotransferase (OR = 5.64, 95% IC 1.7–20.60, p-value = 2.2 × 10^−3^) and aspartate aminotransferase (OR = 11, 95% IC 1.56–77.37 p-value = 1.3 × 10^−3^) suggesting that this null genotype is a risk factor for mild hepatotoxicity induced by carbamazepine^67^. In the same study, neither the dosage of carbamazepine per body weight nor the plasma carbamazepine concentrations were associated with transaminase levels. It is has been suggested that carbamazepine-induced hepatotoxicity was dose-independent and carbamazepine toxicity seems to not be correlated with carbamazepine plasma concentration and that could be rather induced by other factors^67^. In chronic spontaneous urticaria, it has been observed that desloratadine effect depends on GST polymorphism. After treatment, antioxidant status in patients having *GSTM1* null genotype were more improved than those having at least one copy of *GSTM* revealing a better response to desloratadine in homozygous *GSTM1* deletion carrier patients^68^. In Tunisia, in 2012, the mortality rates of tuberculosis was 3 per 100,000 cases^69^. Isoniazid, is used as first-line agent. However, this drug in combination with Rifampicin, are known to cause adverse drug reactions and often impede scheduled treatment and cure and could lead to hepatotoxicity. Homozygosity of *GSTT1* or *GSTM1* null genotypes cause lack of enzyme activity thus leading to the accumulation of the toxic intermediates of Isoniazid metabolisms and hepatotoxins. A statistically significant association between *GSTM1* and *GSTT1* double null genotypes, and the risk of anti-tubercular drug hepatotxicity was found (p = 0.033) between cases and controls^70^. This genetic variable could be used to develop a pharmacokinetic model of isoniazid concentration in order to maximize the probability of achieving its desired therapeutic concentration and avoid it toxicity in the Tunisian population^71^. All these observations suggest that people with these gene deletions might be at increased risk for certain diseases and drug toxicities and alternative therapies may be needed^72^. Therefore, CNVs could be factors to drug over-activation or loss of detoxification activities which might lead to drug toxicities in patients. Consequently, physician could utilize CNVs frequency data to determine the likelihood of their patients experiencing benefits or suffering from drug-induced toxicity. This enables them to take appropriate precautions based on individual genotyping information. Both healthcare providers and national health authorities can leverage this knowledge to guarantee that patients receive the maximum benefit and minimal risk during disease prevention and drug therapy. This approach is largely recognized as the foundation for personalized genomic medicine.

Both our previous and present studies provided insights on the structure of the Tunisian population using in an unprecedented manner CNV data^38^. The use of various scales of genomic polymorphisms captures distinct genomic information. Nevertheless, our findings from population genetic analyses utilizing CNPs and SNPs demonstrate strong agreement as it might be proven by the PCA using both kinds of markers here. The analysis of STRUCTURE shows a high proportion of the European component in the Tunisian population (K = 3) which is in concordance with the PCA analysis suggestion a closer relationship with European than with Africans. This observation confirms our previous finding result based on a correlation analysis of CNV frequency data, thereby providing further evidence^38^. Our population structure analysis also detects a relatedness of the Tunisian population with South Asians and Admixed Americans. These results demonstrated that Tunisian has a significant genetic contribution from Eurasia. This conclusion is based on evidence that the Admixed American population was influenced by recent European contact^73^, supported by other studies^74,75^. For several investigated markers, North African including Tunisian populations exhibit intermediate frequencies indicating a probable ancient and known historical admixture, in addition to genetic drift between European and African populations^34^. Therefore, based on the collective findings, it can be inferred that the Tunisian population could be considered as an admixed one. Furthermore, the results suggest that historical migration waves and invasions did not eliminate or homogenize the genetic diversity but instead contributed to its enrichment.

Advances in genetics have facilitated the reconstruction of human demographic and adaptative history. The removal of rare, harmful mutations from the human population is influenced by demographic and purifying selection, while advantageous variants that enhance survival and reproduction in specific environments can be promoted by positive and balancing selection^76^. Although selection signals for SNP in different ethnic groups have been extensively y explored, such analyses for CNVs are lacking as evidenced by the scarcity of studies using CNV data in the identification of adaptation candidate loci^26,77^. The candidate genes responsible for the signals of selection exhibited a significant enrichment in genes related to drug metabolism, infectious diseases and cancers. These genes could coincide with the environmental and lifestyle attributes of the Tunisian during prehistoric times when the climate was humid and North African were nomadic^78^. These conditions would have influenced the availability and types of foods resources thus affecting nutrition. Variations in the diet, along with changes in food availability due to climate fluctuations, might have influenced the prevalence of malnutrition or diseases. Additionally, the interaction between human and the environment has likely impacted the spread of infectious diseases. Factors, such as proximity to water sources, animal domestication, and contact with other populations could have increased the risk of diseases like zoonoses, vector-borne diseases or waterborne illness. Admixture with other populations could have introduced genetic diversity and potential adaptions to specific environmental challenges. Among the genes assessed as candidate loci for selection adaptation are those of Fc gamma receptors (FCGRs). *FCGR2B* is known to be a resistance factor to malaria and *FCGR3A* is involved in autosomal recessive immunodeficiency. Furthermore, FCGRs have been reported to facilitate Leishmania (L.) internalization especially when in its amastigote form^79^. In Tunisia, zoonotic cutaneous leishmaniasis caused by L. major is considered as significant public health concern (https://www.emro.who.int/neglected-tropical-diseases/countries/cl-tunisia.html). Malaria has also been endemic in the past in Tunisia as it indicated by the geographic distribution and the prevalence of sickle cell disease^80^. Mutations involved in sickle cell disease are well known to be under positive selection conferring an immediate fitness benefit and consequently conferring a resistance to malaria. Other genes, like the FCGRs could have been also been involved in such adaptative phenotypes. *FCGR2B* is a risk factor to systemic lupus erythematous which is an autoimmune disease, and to other bacterial infections including tuberculosis and have also been identified as a candidate loci for selection in our study. As the incidence of the most chronic inflammatory diseases has increased in the industrialized countries, a so called “hygiene hypothesis” has been formulated to justify it^81^. It suggests that the decrease in pathogen diversity in addition to the improvement in hygiene as well as the use of vaccines and antibiotics, were accompanied by a variance in the immune response. Indeed, alleles that helped to fight against infections in the past are now being correlated with higher risks of inflammation or autoimmune disorders^82^. Deschamps et al. suggest that many selective events targeting innate immunity genes could have occurred in a period ranging from 6000 to 13,000 years^83^. All these observations provide further support for the notion that the lifestyle change of the prehistoric human from nomadic life to adopting agriculture and farming in addition to hygiene of the modern human has had have a great impact on the human exposure to microbes and pathogens leading to genetic adaptation of the immune response functions^83^.

The extent to which duplication and deletion CNVs contribute to human genetic diversity, and may or may not convey phenotypes and diseases, is still being unraveled. Throughout our present work, the methods used assumed the neutrality of CNPs, thus, the results presented here have to be taken with caution without further evidences on the functional consequences of CNPs. As more genomes are sequenced, with associated phenotypic and respective environment data, and new analytical tools are developed to detect complete catalogues of CNVs and their breakpoints, we expect to reveal new perspective of gene loss and gain as a pervasive source of genetic change that has great potential to cause phenotypic diversity. The intriguing observation in our present study of numerous gene deletions in apparently “healthy individuals” rises multiple questions regarding the potential adaptability or neutrality of non-functionalization mutations, thus endorsing the “less is more” or “regression evolution” hypotheses^84^. Furthermore, our population genomics investigation could offer the opportunity of assessing processes of gene loss in populations and thus, evaluating the actual gene dispensability. In this regard, the population gene dispensability concept could be considered and could pave the way to identify candidate gene losses for which non-functionalization is adaptive and consequently having possible relevance in biomedicine^84,85^.

## Conclusion

In summary, we conducted a comprehensive population genome-wide analysis of copy number polymorphisms of the Tunisian population comparing it with 11 HapMap III populations. This investigation has extensively expanded our understanding of these markers. We explored the distribution and diversity of CNVs at the population level, providing insight into the genetic relationships between the Tunisians and different other populations for the first time using CNP markers. Additionally, we have also annotated Tunisian high-frequent CNPs and uncovered several putative candidate genes that may have undergone selection. The CNP features and population genetic structures confirm previous findings. The distinctive genomic structure of the Tunisian population could have been shaped by various forces such as natural selection and genetic drift, leading to the emergence of singular genomic variants involved in specific biological processes. Consequently, our study provides a foundation for prioritizing population specific genome organization and revealed genome elements that have been essentially uninvestigated. Conducting additional research on North African populations could offer more insights on the CNVariome of these populations, enhancing our comprehension of the human genome evolution and its medical implications.

## Methods

### Samples genotyping platform and CNP calls

A total of 102 healthy individuals from Northern, Central and Southern Tunisia (TN) have been recruited and analyzed in our previous study^38^. Informed consent was obtained from all the participants and no personal identifiers were used to maintain participant anonymity. Following the principles of the Declaration of Helsinki Principles, we obtained the ethical approval from the biomedical ethics committee of Pasteur Institute of Tunis (PV09/06, IRB# 0000000044). Theses 102 Tunisian individuals underwent genome-wide scanning using the Affymetrix Genome-Wide SNP Array 6.0 as mentioned in a previous study^86^.

In addition, raw Affymetrix SNP Array 6.0 of individuals from the HapMap Project Phase III have been downloaded from the ftp site (ftp://ftp.ncbi.nlm.nih.gov/hapmap/raw_data/hapmap3_affy6.0/). The studied populations included people from various regions: African ancestry in the southwestern USA (50 ASW), the Luhya in Webuye, Kenya (83 LWK), the Maasai in Kinyawa, Kenya (147 MKK), the Yoruba in Ibadan, Nigeria (120 YRI), the Utah residents with Northern and Western European ancestry (115 CEU), the Tuscans in Italy (83 TSI), the Chinese community in Metropolitan Denver, Colorado, USA (85 CHD), Gujarati Indians in Houston, Texas, USA (72 GIH), the Japanese in Tokyo, Japan (91 JPT), the Han Chinese in Beijing, China (89 CHB) and Mexican ancestry in Los Angeles, California, USA (56 MEX). Only unrelated individuals were included in our analysis leading to a total of 1093 unrelated healthy samples from the 12 populations analyzed.

### Copy number polymorphism (CNP) identification using Canary

The Canary algorithm of the Birdsuite software was applied on the 102 Tunisian individuals to identify the CNPs^38^. This algorithm calls simultaneously across multiple individuals at pre-defined genomic positions^87^. Similarly, for the 1316 CNPs from the 11 HapMap phase III project populations, their genotypes were identified using the same canary algorithm and the same parameters as in our previous study^38^. We specifically focused on CNPs located on the 22 autosomes (1279 loci) for subsequent analysis considereing those with integer copy numbers called with high confidence according the software recommendation (confidence score > 0.1) and differing from the neutral copy state. The CNPs were classified into three classes “deletion”, “duplication” and “mixed” which are loci encompassing both deletions and duplications as previously reported^38^. These are qualified as allelic CNPs (loci with copy number states differing from the normal copy number state (CN = 2)).

We filtered CNPs according to their size, retaining only those with a size greater than or equal to 1 kb. In addition, in order to provide reliable data, the 1000 Genomes phase 3 structural variants data have been downloaded as reported in the original publication in VCF format^12^; https://www.internationalgenome.org). We compared our CNP data of each population while requiring a minimum of 50% of reciprocal overlap size using Bedtools v2.2.25.0^88^. CNPs with frequency greater than 90% not overlapping with any segments from the 1000 Genomes dataset were considered as potential false positive and therefore the have been removed. Genomic coordinates for CNPs were mapped to the assembly build 37 of the human genome (hg19).

### Population comparisons for features and integer copy numbers of the CNPs

We studied the distribution of CNP categories, as well as the size and frequency features using the Chi-square and Wilcox tests to compare the Tunisian population and each of the HapMap populations. Furthermore, we examined population differences in the integer copy numbers using the Fisher’s exact test. All the comparisons have been performed as a pairwise procedure between the Tunisian population and each of the 11 HapMap III populations using the R programming language and statistical software. CNPs resulting from copy number comparisons with p-value less than 5% were considered for further annotations, with p-value adjusted using the FDR (false discovery rate) method.

### Population structure and differentiation

The CNP calling results by Canary included 7 copy states (0–6): 0-copy state (homozygous deletion or CN = 0), 1-copy state (heterozygous deletion or CN = 1), 2-copy state (normal state or CN = 2), 3-copy state (single copy duplication or CN = 3), 4-copy state (double copy duplication or CN = 4), 5-copy state and 6-copy state (amplification or CN = 5 and CN = 6, respectively). Such copy state results can be explained by a four-allele system: 0 copy-allele for loss allele, 1 copy-allele for normal allele, 2 copy-allele and 3 copy-allele for gain-allele. Therefore, we coded biallelic CNPs with genotype ‘0, 1, 2, 3, 4, 5 and 6’ as ‘0/0, 0/1, 1/1, 1/2, 2/2, 2/3 and 3/3’ for the pair of two autosomal chromosomes.

Tunisian high-frequent CNPs were defined as deletions or amplifications with significantly high frequencies in the Tunisian population determined by Fisher’s exact test, with p-value adjusted using the FDR method.

We used principal component analysis (PCA) to infer population structure that was performed with the “dudi.pca” function implemented in the R package “ade4”^89^ to compare the Tunisian population with the HapMap III populations using the CNP and SNP data. The PCA plots were visualized with the “factoextra” package^90^. Ellipses are added to the PCA plots to represent confidence regions around groups of observations, thus, helping visualization of the spread and dispersion of data points in the principal component space for different groups. In addition, the R package “Hierfstat”^91^ was used to assess the genetic structure similarity degree between the various ethnic populations analyzed in this study using the same CNP data. The pairwise Fst values have been calculated using the “pairwise.WCfst” function of the hierfstat package. The differentiation magnitude among geographic population has been assessed with values as following: 0 < Fst < 0.05: weak differentiation; 0.05 < Fst < 0.15: moderate differentiation; 0.15 < Fst < 0.25: high differentiation and Fst > 0.25: very high differentiation.

We further performed population clustering using STRUCTURE 2.3.4 software^92^ which implements a Bayesian clustering algorithm to assign samples within a hypothetical group. The algorithm underwent 24 runs for the scenario 2–11 clusters (K = 2–11) in our data set, each run consisting of 10,000 iterations after a 10,000 burn-in period. Optimal K was estimated using the ∆K method and by plotting the likelihood of K for each value of K using the “pophelper” R package^42,93^. We performed an alignment of assignment clusters across replicate runs with CLUMPP^94^ using the default parameters (LargeKGreedy algorithm, random input order and 2000 repeats). DISTRUCT was used to visualize the output^95^.

We also presented a measure to characterize the genetic difference of CNP genotypes between two individuals for the studied loci. An Euclidian genetic distance matrix between two samples from two populations was calculated. Based on this matrix, we build a phylogenetic tree by UPGMA with 1000 bootstrap replications. The phylogenetic analysis was performed using the “poppr” R package^96,97^.

### Selection signature and gene enrichment analysis

For all the studied populations, we calculated the CNP allele frequencies. Then, we calculated the pairwise Fst values for each marker by comparing the Tunisian population with the other HapMap populations. The Fst values for each CNP segments were determined using the equation described previously^98^. Subsequently, we ranked the CNPs according to their Fst values for each population pair. The top 1% of CNPs in the Tunisian population were identified. We took also into consideration the population-specific CNVs (Supplementary Table 10). According to this Fst value, CNPs segments were retrieved to discover CNV-overlapping genes potentially related to positive selection.

### Functional annotations

The Tunisian high-frequent CNPs were annotated using the AnnotSV software which is designed for annotating and ranking structural variants providing access to the ACMG classification OMIM and structural variation databases^99^. We also used the PharmaGKB database (https://www.pharmgkb.org) to check if genetic variations in the genes harboring Tunisian high-frequent CNPs have been already associated to drug response. Only genes with evidence of association are reported. The selected signatures were also annotated. The candidate genes were examined for functional enrichment of KEGG and GO pathways using the DAVID bioinformatic resource (https://david.ncifcrf.gov). The pathways and GO terms exhibiting p-values < 0.05 adjusted for FDR were considered as significant.

### Statistical analysis

All the downstream analysis were performed using the statistical software R (http://www.r-project.org). The visualization was performed with the ggplot2 R package^100^.

### Ethical approval

Informed consent was given by all the participants. No personal identifiers were used and participant identities were kept anonymous. According to the Declaration of Helsinki Principles, we obtained the ethical approval from the biomedical ethics committee of Pasteur Institute of Tunis (PV09/06, IRB# 0000000044).

### Supplementary Information

Supplementary Information.

Supplementary Tables.

## Data Availability

The datasets substantiating the article's conclusions can be found in the article itself, Supplementary Tables, and Supplementary Data. Additional supporting data are available from the corresponding author upon reasonable request. As genetic data in Tunisia are considered personal and private, we have included the minimal dataset as supporting files. However, full raw data cannot be publicly submitted due to regulations. Interested researchers may request access to the full raw data through the corresponding author and with the approval of our IRB.
