# Supplementary material for: Ethnic and functional differentiation of copy number polymorphisms in Tunisian and HapMap population unveils insights on genome organizational plasticity
Source: Sci Rep. 2024 Feb 26;14:4654. doi: 10.1038/s41598-024-54749-8 (PMC10897484; doi:10.1038/s41598-024-54749-8)

Supplementary file 1: STRUCTURE plots for K2,K4-11

K2:

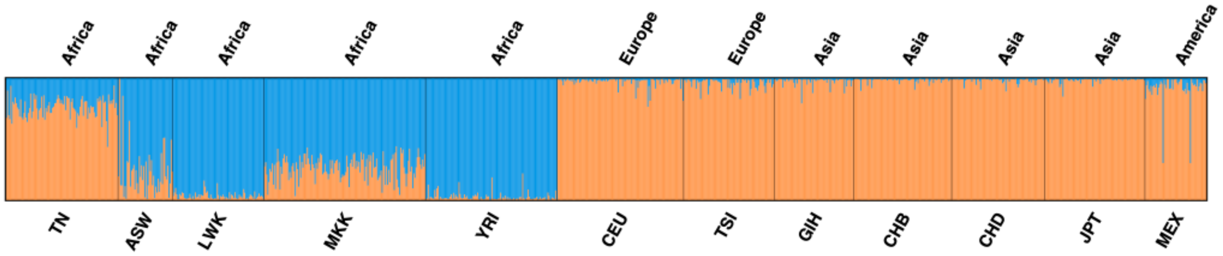

K4:

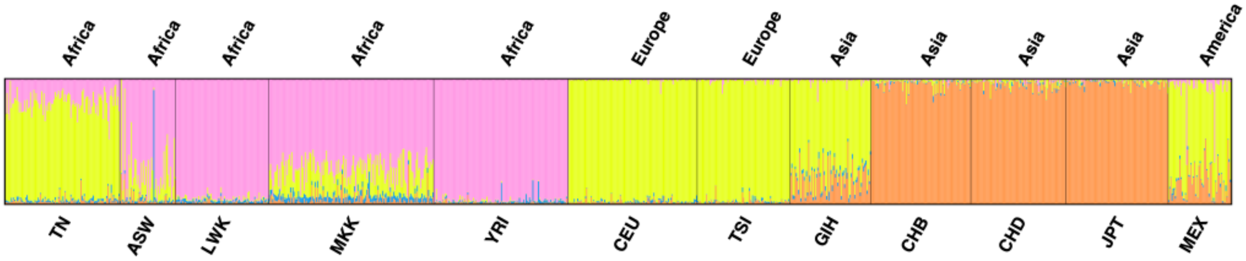

K5:

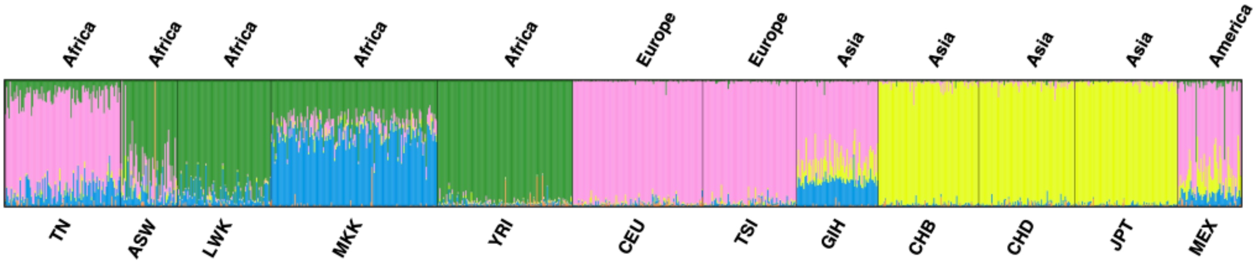

K6:

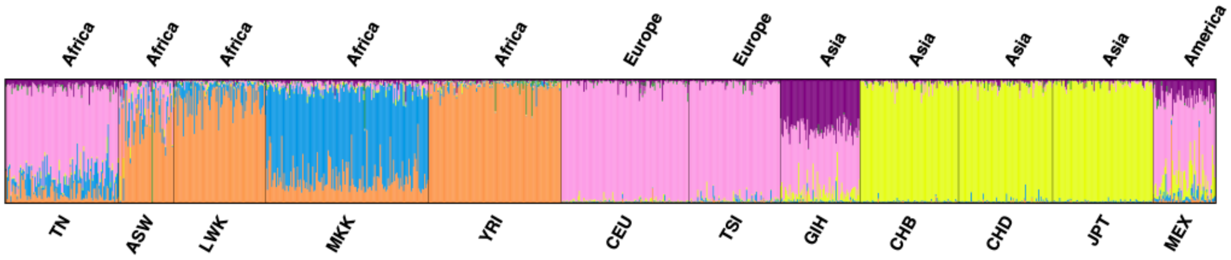

K7:

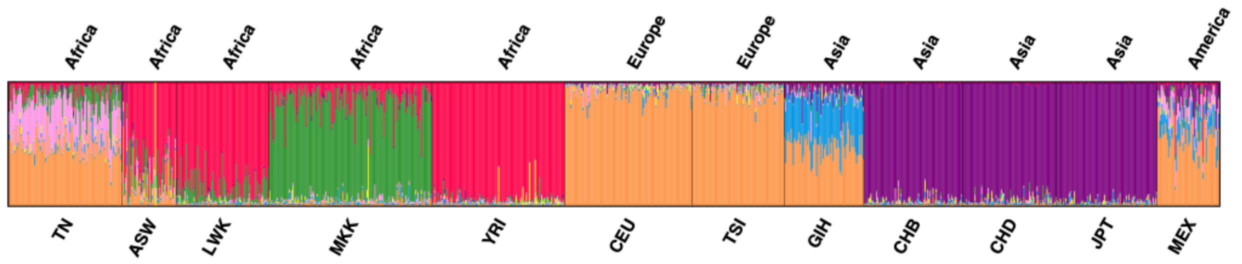
Africa
Africa
Africa
Africa
Africa
Europe
Europe
Asia
Asia
Asia
Asia
America
TN
ASW
LWK
MKK
YRI
CEU
TSI
GIH
CHB
CHD
JPT
MEX

K8:

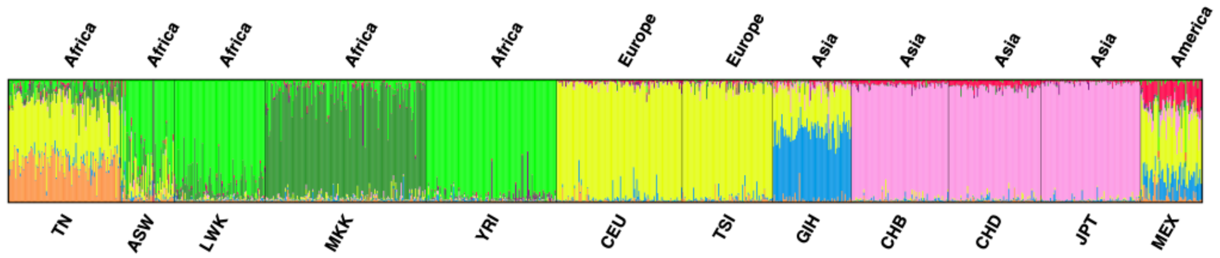
Africa
Africa
Africa
Africa
Africa
Europe
Europe
Asia
Asia
Asia
Asia
America
TN
ASW
LWK
MKK
YRI
CEU
TSI
GIH
CHB
CHD
JPT
MEX

K9:

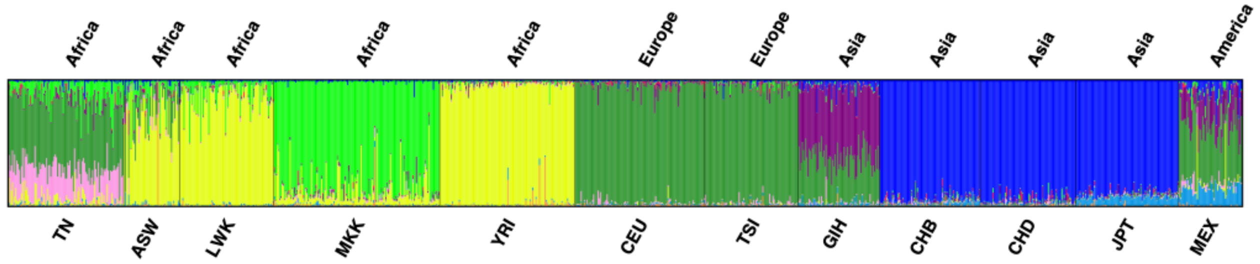
Africa
Africa
Africa
Africa
Africa
Europe
Europe
Asia
Asia
Asia
Asia
America
TN
ASW
LWK
MKK
YRI
CEU
TSI
GIH
CHB
CHD
JPT
MEX

K10:

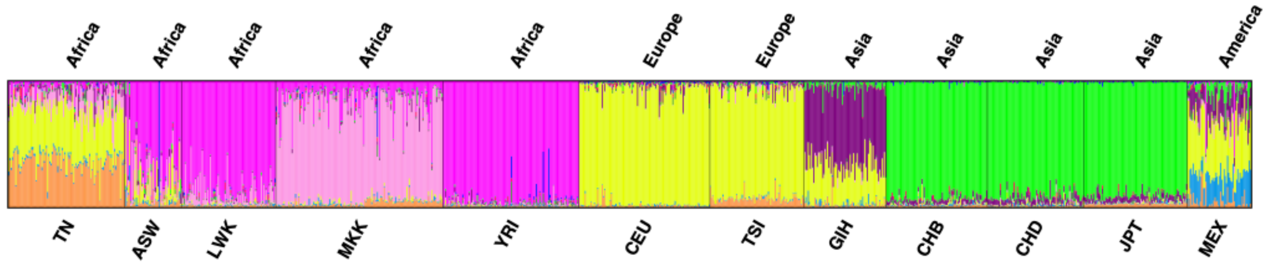
Africa
Africa
Africa
Africa
Africa
Europe
Europe
Asia
Asia
Asia
Asia
America
TN
ASW
LWK
MKK
YRI
CEU
TSI
GIH
CHB
CHD
JPT
MEX

K11:

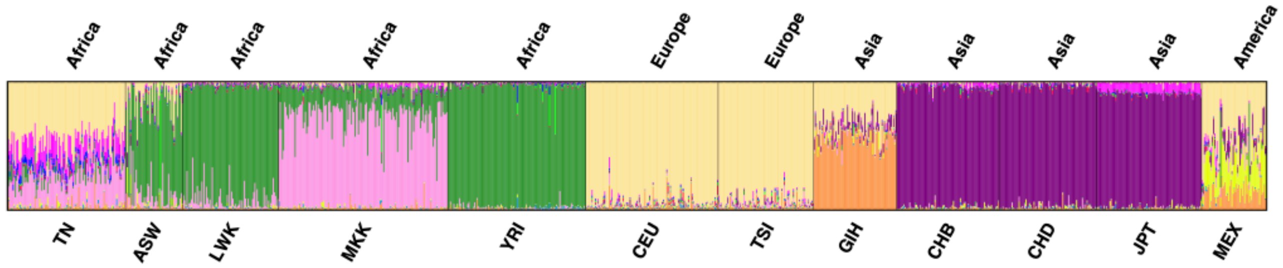
Africa
Africa
Africa
Africa
Africa
Europe
Europe
Asia
Asia
Asia
Asia
America
TN
ASW
LWK
MKK
YRI
CEU
TSI
GIH
CHB
CHD
JPT
MEX

Supplementary file 2: UMAP analysis using the CNP data in the 12 studied populations

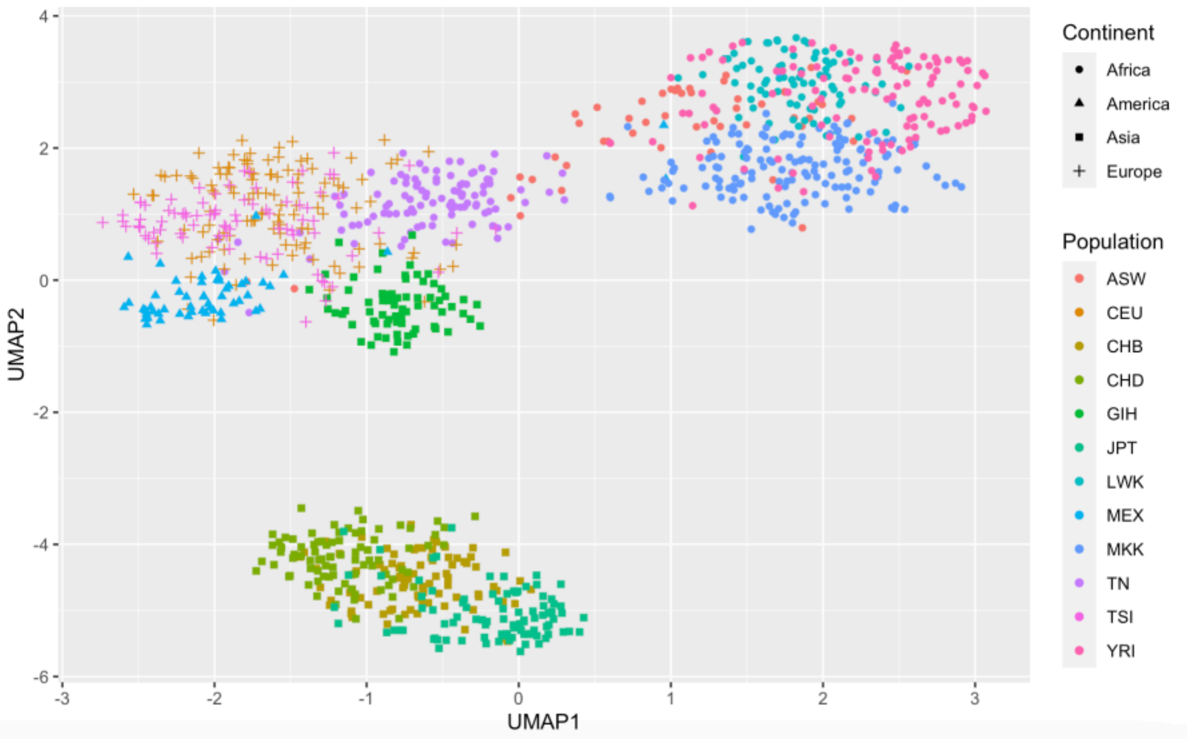

Supplement: Supplementary file 1 — Supplementary Information. [file 41598_2024_54749_MOESM1_ESM.pdf]
